# Honesty in Human Subject Research

**DOI:** 10.1007/s11673-024-10357-9

**Published:** 2024-07-24

**Authors:** Sungwoo Um

**Affiliations:** Department of Ethics Education, Seoul National University, 1, Gwanak-Ro, 11-411, Gwanak-Gu, 08826 South Korea

**Keywords:** Human subject research, Deception, Honesty, Autonomy, Debriefing, Right not to be deceived

## Abstract

In this paper, I discuss the ethical issues related to deception in human subject research in terms of honesty. First, I introduce the background and suggest the conception of honesty that understands it as involving respect for the right not to be deceived (RND). Next, I examine several ways to address the ethical issues of deceptive elements in the human subject research and show why they fail to adequately meet the demand of honesty. I focus on how to make an honest research plan and examine after participation and before participation phases in turn. Then I conclude by suggesting possible strategies to minimize dishonesty in human subject research.

## Introduction

Deceiving a person is generally regarded as morally objectionable since it is a common way of disrespecting a person. Deception in human subject research is particularly problematic because research aims at generating knowledge rather than benefiting the participants and participants have limited control over the risks they are exposed to (Athanassoulis and Wilson 2009). Thus, informed consent and transparency are now fundamental principles in conducting ethical human research. However, many studies that recruit human subjects involve some forms of deception for prospective scientific, educational, or other applied values.

In this paper, I discuss the ethical issues related to deception in human subject research in terms of honesty. While there have been attempts to address the ethical issues involved in deceptive studies, they have focused on identifying the harms of deception, examining how to minimize the element of deception itself, or finding justifying conditions for deception in human subject research (e.g., Wendler 1996; Wendler and Miller 2008; Athanassoulis and Wilson 2009). However, what is ethically more important is not whether the given research involves deception per se but whether it manifests the vice of dishonesty. This is because the normative judgment about an instance of deception can be changed depending on whether it manifests dishonesty or not, or so I shall argue. Honesty as a virtue is not the same as a mere disposition to refrain from deception. If so, we need to discuss the ethics of deceptive research in terms of honesty, which I understand as involving respect for the right not to be deceived (RND).

How would an honest researcher make a plan for a study when it seems to require deception for its scientific validity? This is the main question I will address in this paper. First, I introduce the background and suggest the conception of honesty that understands it as involving respect for the right not to be deceived. Next, I examine several ways to address the ethical issues of deceptive elements in the human subject research and show why they fail to adequately meet the demand of honesty. I focus on how to make an honest research plan and examine after participation and before participation phases in turn. Then I conclude by suggesting possible strategies to minimize dishonesty in human subject research.

To begin, see the following four conditions suggested by the Ethical Principles of Psychologists and Code of Conduct produced by the American Psychological Association (2017):

1. The use of deception is justified by the study’s significant value.
2. Any equally effective, nondeceptive approaches are not feasible.
3. Deception is not reasonably expected to cause physical pain or severe emotional distress.
4. Any deception is explained to participants, preferably at the conclusion of their participation, but no later than the conclusion of the research, and participants are allowed to withdraw their data.

With regard to the research discussed in this paper, I assume (2), (3), and (4) are met. I further assume that research participants do not expect to receive any benefits from the participation and that the purpose of the research is itself socially valuable and morally unobjectionable. In relation to (1), I assume that it may be possible that sometimes deceptive research is ethically permissible, all things considered. As being honest is not the only ethical consideration, deceptive research may be justified by its significant prospective scientific, educational, or other applied values. The aim of this paper is not to argue that no deception is ethically permissible in any human subject research. Rather, its aim is to examine the deceptive elements in research that manifest dishonesty and seek the ways in which we can minimize them when possible.

## The Difficulties of Deceptive Human Subject Research

Let me begin with some examples of deceptive human subject research. First, psychologists deceive participants about the methods and/or aim of an experiment when accurate disclosure would undermine the validity of the results. Milgram’s obedience experiment is a famous example. In this experiment, Milgram was able to collect participants’ honest responses to the authoritative figure’s order by concealing the fact that the button they push does not actually give any electrical shock to the confederates (Milgram 1974). Some medical research also involves deceptive elements in its process. For example, some clinical trials might involve a degree of deception. Participants could be misled about the true nature of the drug being tested, its potential side effects, or the likelihood of receiving a placebo. This is done to ensure unbiased reporting of effects and to study the placebo effect. Educational studies may also involve deceptive practices by providing false information or feedback to students. For instance, students could be given false scores or feedback about their performance to study how it affects their motivation or learning strategies.

Such deceptive human subject research has an inherent difficulty. In some types of research, on the one hand, deception is crucial for acquiring “natural” results that have scientific validity. On the other hand, it is ethically important to get informed consent to participate in the research from the subject. The act of asking for informed consent is a gesture of respecting the participants as persons. However, deception does not allow this possibility because it presupposes that the potential victim does not know that she will be deceived and what exactly she is to be deceived about. When it comes to other forms of harms, we can give informed consent to be harmed in a described way in advance. For example, one can consent to allow the researcher to take one’s biopsy. In contrast, it is hard, if not impossible, for one to give *informed* consent to be deceived about a particular matter in advance, since in most cases the very awareness of the deception frustrates the purpose of deception.^1^ There are two main features in deception that make it hard to address the ethical issues related to deception. The first is *irreversibility*: once the deception takes place, it is hard, if not impossible, to undo it. The second is *opaqueness*: the subject’s willingness to participate is in principle opaque to the consent-seeker unless she explicitly asks for the consent. Given such difficulties, addressing the ethical issues concerning deception is becoming increasingly important for researchers, especially those who work in the fields of human subject research such as psychology and medicine.

However, whether something is deceptive is not as important as whether it manifests dishonesty or not. For, I believe, despite the appearance, deception per se is not sufficient to render an action morally objectionable. What normatively matters is whether the case of deception meets the demands of *honesty* or not, since not all cases of deception manifest dishonesty. To evaluate a case of deception, we need to see the reasons for deceiving and take all the normatively relevant elements in question. Thus, in this paper, I analyse the cases of deceptive research in terms of honesty rather than merely whether the deception is involved or not.

Moreover, there are other reasons to care about honesty in human subject research. First, it is important for the researcher’s (or other confederates such as assistants) cultivating an honest character as well as avoiding moral distress that comes from being dishonest. For example, it is reported that the confederate “who was involved in the [deceptive] experiment was, and continues to be, doubtful and guilty about their part in the study” (Oliansky 1991, 256). Also, there is a need to (re-)gain and maintain trust by developing and communicating the researcher’s honesty to the (potential and actual) subjects. The advancement of human subject research heavily relies on the trust of the public, including potential research participants. Insofar as potential participants of research tend to deem the researchers dishonest, they would become much less likely to contribute to the research by participating in the studies. For these and other reasons, it is important for researchers to be honest in making a research plan and executing it.

## Honest Research Plan

Then, what does it take to be honest? I suggest that honesty’s moral ground is the *respect for the right not to be deceived* (RND). Thus, an honest researcher would respect the subject’s RND at least to the extent that the circumstances allow. Let me briefly explain the ground for RND. We have a basic interest in *being in touch with reality* and a minimal claim that others do not positively intrude into our pursuit of truth by deceiving us, at least if there is no good reason to do so.^2^ Moreover, there is an additional ground for the (potential) research subject’s right not to be deceived by the researcher about the information relevant to the research (e.g., research purpose, methods, risks, and benefits, etc.). Thus, in my view, honesty requires the researcher to respect the subject’s RND.

Before proceeding, let me add a few notes of clarification. On my account, *respecting* someone’s RND involves more than merely refraining from infringing on her RND. For one could easily refrain from doing so for some non-moral reasons such as those of self-interests. An honest person would refrain from infringing on someone’s RND out of respect for it. Also, dishonesty is not simply a matter of infringing on someone’s RND, since one may do so for a justifiable reason (e.g., to save someone else’s life).

Moreover, although deceiving is a type of action that a dishonest person would often perform, deception itself does not directly manifest the agent’s dishonest character. Above all, deceiving someone does not in itself imply disrespecting her RND, since one may deceive someone not due to disrespect for her RND but due to more important considerations—e.g., to save an innocent person’s life—that demands one to use deception as the only option available to do the right thing.

I believe that RND is not absolute in that it can be overridden by some other considerations—e.g., saving an innocent person’s life or making some urgent medical discovery. However, even if one deceives someone else, it does not necessarily mean she is thereby manifesting dishonesty insofar as the deception is because of the demand of more important considerations rather than because of the lack of due respect for the subject’s RND. The aim of this paper is to find ways to minimize the elements of dishonesty in research by analysing the considerations relevant to honesty.

With this conception of honesty in mind, let me now examine existing or suggested ways to address the ethical issues regarding deception in human subject research. I will consider the research plan from the perspective of honesty and divide it into two phases: after participation and before participation.

### After Participation

There are possible attempts to make up for the deception involved in the research *after* participation, such as compensating, expressing fitting attitudes, and debriefing. I do not deny that those attempts may at least alleviate the ethical objectionability when the deception has already been committed. But what I am trying to argue in this section is that an honest researcher would not make a deceptive research plan as if those ex post measures will suffice to “undo” the wrongs and harms committed to the deceived participants. Let us examine each measure in turn.

#### Compensation

First, there are attempts to ethically make up for the deception in research by offering the subject some sort of *compensation* (e.g., monetary or material rewards) after the participation. Suppose that researchers of a deceptive psychological study try to make an extra payment for the deceptive element involved in the research. However, can deception manifesting dishonesty be compensated in this way? Insofar as the deception manifests disrespect for the deceived subject, this disrespect cannot just be compensated away by offering *ex post* reward. Disrespecting someone’s RND would be a case of *wronging* that person, as opposed to merely *harming* her unless there are overriding moral considerations. A wrong committed to a person is not simply neutralized by some benefits given to that person for the purpose of offsetting the harms done. Thus, a deceptive research recruitment may be wrong even if the subject participates in the given research after being informed and warned about the risks of being harmed, unless she is also warned about the risk of being *wronged* by deception.

Furthermore, sometimes the attempt to compensate for an intentional wronging can be even more insulting to the victim. It would be seriously disrespectful to treat another person as if she is a kind of being to whom a wrong committed to her can be counterbalanced simply by offering some benefits. Such an expectation would itself manifest an attitude degrading the status of the person in question. Thus, insofar as the deception has happened by design, rather than by accident, compensating or even trying to do so would not be an appropriate way to address the ethical issue related to the deception in research.

#### Fitting Attitudes

Second, the researcher may try to make up for the deception by communicating some sorts of *fitting attitude* to the subject. For example, one may apologize, seek forgiveness, or express gratitude to the subject for her being the victim of the deception involved in the research. However, such attitudes do not seem to render the researcher adequately honest, either.

First of all, apologizing after conducting the deceptive research in question does not manifest respect for the subject’s RND as it is to be deem insincere. Doing what one would apologize for later shows that one values doing what one plans to do over being honest to the subjects anyway. The very fact that the researcher deceived the subject on purpose shows that the apology is not sincere. This kind of attitude suggests that the researcher would be willing to involve deception in her research again if that is necessary for its scientific validity provided that she gives ex post apology.

Seeking forgiveness faces a similar difficulty. It is generally easier to ask for forgiveness after wronging someone than to ask for permission to wrong her in advance. In fact, if one gets permission for the given kind of wronging, it may no longer be “wronging.” To give an analogy, taking someone else’s money *without* permission is an obvious case of wronging—we call it “stealing”—doing the same thing *with* permission is not—we call it “borrowing.” Similarly, depriving the victim of the opportunity to make her own choice may make the activity that would have been permissible wrong. This is the point of seeking informed consent in most human subject research before recruiting subjects. But seeking forgiveness afterward is certainly different from seeking permission in advance, since it does not allow the victim the room for declining.

Even if the victim ends up forgiving the researcher for deceiving her without permission, it would be because of her generosity rather than because the researcher *deserves* forgiveness or the research itself is ethically designed. Even if the participant refuses to forgive the researcher for deceiving her, the researcher would not have legitimate grounds to blame her or complain about it, as the participant is wrongfully deceived and thus has a sort of right not to forgive.

Lastly, expressing gratitude to the deceived subject is insufficient to address the ethical concern about the deception as well. The subject of deceptive study does not have the opportunity to participate in it voluntarily because the true options that would have been available to her are concealed by the deception. For example, a person who has participated in what she believed to be research about measuring her cognitive ability might not have participated if only she was informed in advance that it is actually about measuring her disposition to show dishonest behaviours. Gratitude is not fitting to an involuntary victim. This is why it is not fitting for a thief to thank the victim after stealing. Similarly, it would be absurd to express gratitude to the subject for participating in deceptive research, especially one that she would not have participated if only she had known about its deceptive elements. For these reasons, it is insufficient to apologize, seek forgiveness, or express gratitude after wronging a victim intentionally with deception.

#### Debriefing

Let us now consider *debriefing*, which is perhaps the most common method that researchers use to address the ethical issues related to the deception in research. Debriefing is generally recommended as a measure to take to address the ethical concerns that may arise from deception involved in human subject research:

[E]xplain any deception that is an integral feature of the design and conduct of an experiment to participants as early as is feasible, preferably at the conclusion of their participation, but no later than at the conclusion of the data collection, and permit participants to withdraw their data.

Ethical Principles of Psychologists and Code of Conduct (2017)

[W]henever appropriate, the subjects will be provided with additional pertinent information after participation.

The U.S. federal regulations (DHHS 2018, 45 CFR 46.116(f)(3)(v))

It is said that debriefing has the following purposes:

(a) a dehoax, meant to remove false beliefs and give participants the truth they are owed; (b) a desensitization, meant to address participants’ distress; (c) a justification of the use of deception, meant to hold investigators accountable to participants; and (d) an opportunity to withdraw data, meant to restore some autonomy that was violated. (Sommers and Miller 2013, 104)

However, debriefing does not adequately address the problem of dishonesty, either. This is mainly due to the irreversibility of deception. One cannot retrospectively give consent to the deception that has already happened. Debriefing is neither a way of respecting the subject’s RND nor a way of making up for the disrespect to her RND, insofar as she has been intentionally deceived. But here we are discussing only the cases in which the deception in question is a constitutive part of the research *plan*. Thus, even when one is debriefed about the information that reveals the deceptive nature of the research—e.g., the true purpose of the study, research methods, risks, etc.—it cannot restore the deprived opportunity to autonomously choose whether to participate in thus understood research based on adequate relevant information.

It is true that the participants can be given the opportunity to withdraw data gained through deception after the debriefing. Still, it does not restore the opportunity *not to participate* in the study at the beginning. The options one can choose after the debriefing may include understanding, forgiving, or tolerating; but consenting or declining is not one of them. When the subject is already deceived by the researcher on purpose, no debriefing can reverse the disrespect paid to the subject’s RND. This is why debriefing is not sufficient to keep the research honest.

Of course, I admit that it is important for researchers to communicate with the participants during and after their participation in the study and that debriefing can be one important way of communicating with them about the nature of the study. What I am saying here is just that such a communication is not an ethical neutralizer of the pre-designed deception in a dishonest study.

### Before Participation

We have examined some possible ways to address the issue of dishonesty *after* the subject’s participation. I have shown some reasons why those retrospective attempts may be insufficient to make the research plan to meet what honesty demands. Now it is time to consider possible attempts to steer clear of dishonesty in research *before* the subject’s participation.

#### Reasonable Expectation

One important consideration in making an ethical research plan is to *reasonably expect* different aspects of the results before recruiting participants or conducting the research. Honest researchers would make the research plan based on a reasonable expectation of the deception’s possible harms (e.g., mental distress) on the subjects or the likelihood that the deception would affect their willingness to participate. See the following codes and regulations:

[D]o not deceive prospective participants about research that is *reasonably expected* to cause physical pain or severe emotional distress. (Ethical Principles of Psychologists and Code of Conduct 2017)

In order for an IRB to waive or alter consent as described in this subsection, the IRB must find and document that:… (iv) the waiver or alteration will not adversely affect the rights and welfare of the subjects[.] (The U.S. federal regulations (DHHS 2018, 45 CFR 46.116(f)(3)(iv))

Some commentators seem to believe that an honest research plan requires reasonable expectation of what kind of deception would affect the subjects’ willingness to participate. For example, Wendler and Miller argue:

IRBs should consider allowing deceptive research only when… (3) subjects are not deceived about aspects of the study that would *affect their willingness to participate*, including risks and potential benefits. (Wendler and Miller 2004, 600)

However, a research plan that involves deception about aspects that are reasonably expected not to affect the subjects’ willingness to participate may still fail to avoid the charge of dishonesty. Suppose that the researcher tells the subject, “We deceived you about X because, according to our judgement, whether you are deceived about X would not affect your willingness to participate.”

Let us consider two possible scenarios. In Scenario 1, it turns out that the subject finds the aspect about which she is deceived in the research is very important to her and says that she would never have participated in the research if only she knew that it involved such kind of deception. In such a case, the researcher (or IRB members) cannot claim that the research plan was still honest just on the ground that their expectation of the subject’s response was—though not quite correct—*reasonable enough*. That is, the researcher cannot blame the subject for not being reasonable by saying something like, “Most people would not take issue with or be emotionally distressed by such a trivial deception!” or “‘A majority of subjects said they are willing to participate again in the given deceptive study!” (Fleming et al. 1989).

One important reason why the researcher should ask for the subject’s informed consent is to respect the subject’s autonomy. What is important with regard to respecting the subject in the research plan is not to make an *epistemically reasonable* expectation of the subject’s response. Rather, it is to respect this particular individual’s autonomy by asking her if *she* is—as opposed to any “reasonable” person would be—willing to participate in the study. Thus, even if it is true that most people would not have changed their willingness to participate due to the kind of deception involved in the given subject, it does not make the research honest at least in relation to the given particular subject who would find the deception objectionable.

The potential victim might “unreasonably” have declined the proposal to be deceived without any good reason. Still, the subject does not have to have any reasonable ground for her decline to justify her choice insofar as she has the right to decide whether or not to enroll in the study at her will. Thus, when a particular subject refuses to give consent to or feel distressed by what most people are expected to consent to and feel no distress about, anyone who respects this subject just should respect her own decision regardless of its alleged reasonableness.

Here we need to be careful in talking about the ‘reasonable person’ standard in relation to deceptive research. Researchers generally need the reasonable person standard because it is practically inefficient, if not impossible, to give all the information that the candidate (may) wants to know. However, deceptive research is a special case. Suppose that a particular participant later realized that some information—say, the colour of walls in the lab—was not given before participating. Suppose further that this is a *relevant* piece of information in the sense that she would not have consented to participate in the experiment if only she were informed that the colour of the wall was pink (She hates pink!). But suppose that the researcher did not reveal this information only because she thought—by the “reasonable person” standard—the colour of the wall would not be relevant for informed consent. In such a case, there is no issue of dishonesty because this omission was not due to the researcher’s lack of respect for her RND.

In contrast, suppose that the researcher intentionally omitted giving a participant just based on the assumption that a reasonable people would not care about a deception about such a “trivial” matter. If so, if it turns out that the participant is unusually sensitive to being deceived (in general or about certain particular matters), then the researcher cannot avoid the charge of dishonesty simply by saying, “No reasonable person would take issue with such a trivial matter!”.

Let us now consider Scenario 2. In this scenario, it turns out that the deception does not negatively affect the subject’s feelings or willingness to participate. However, this fact may not necessarily let the researcher avoid the charge of dishonesty either insofar as she still fails to respect the subject’s RND.

First, a researcher may disrespect the subject’s RND without disrespecting her right to *autonomy*. RND is to be distinguished from the right to autonomy, since one may violate it without violating the right to autonomy when one deceives someone about a matter that she does not have any practical interest. If the researcher deceives the subject about the information that would counterfactually affect her willingness to participate in the study, then it seems to be a clear case of disrespecting her right to autonomy. But if the deception turns out not to affect the subject’s willingness to participate, it may be claimed, her right to autonomy is not disrespected at least in this regard.^3^ Even so, it does not mean that this “counterfactually innocent” deception is free of the charge that it still disrespects the subject’s RND.

Even if the deception does not disrespect the subject’s right to autonomy by counterfactually affecting her choice, it may still disrespect her RND since it can be violated even if it does not affect the subject’s willingness to choose her own action. For instance, one may have RND about the amount of money in one’s bank account even if she does not intend to do anything with that money.^4^ Regardless of whether deception involved disrespect for the subject’s autonomy or manipulation of the subject, it still may be ethically objectionable insofar as it manifests the vice of dishonesty against the subject. Thus, to examine the ethical permissibility of a deceptive study, we should examine whether the deception manifests any disrespect to the subject’s RND, not just its expected impact on a reasonable person’s willingness to participate.

It is also important to note that whether the subject finds the deception objectionable is not directly related to whether her RND is disrespected. A right may be disrespected even when the right-holder is not aware of the violation or does not feel any discomfort or emotional distress when one finds the fact that one’s right is violated or disrespected. For example, a person who has a servile character or low self-respect may believe that she does not has any human right. But such a false belief about one’s own right does not make others’ disrespect for or violation of her human right ethically permissible. Similarly, the permissibility of a deceptive study depends on whether it involves objectionable kind of disrespect for the participant, rather than on how she subjectively takes it. In this sense, disrespecting the participant’s RND can be ethically problematic regardless of how she subjectively experiences it.

#### Consent for Deception

One obvious way to avoid dishonesty is to show respect for the subject’s RND by asking for the subject’s consent to participate in the study *after* informing her about the deception. A researcher can do it by asking the subject to waive her RND in relation to the deceptive element of the study before she consents to participation. However, as mentioned above, informing the subject about the details of deception before participation may undermine the study’s scientific validity by failing to obtain the subject spontaneous and “candid” response. This is not an easy task because the process of asking for the informed consent should be transparent enough not to violate the subject RND and at the same time opaque enough not to undermine the main purpose of the study or its scientific validity.

Dave Wendler and Franklin G. Miller offer a promising solution, which they call *authorized deception*:

You should be aware that the investigators have intentionally misdescribed [or left out information about] *certain aspects* of this study. This use of deception is necessary to conduct the study. However, an independent ethics panel has determined that this consent form accurately describes the major risks and benefits of the study. The investigator will explain the misdescribed aspects of the study to you at the end of your participation.

(Wendler and Miller 2008; see also Wendler 1996)

I believe asking for consent about the possible deception is one of the most promising ways to avoid the charge of dishonesty. However, the form suggested above still does not seem adequate. Even if the subject is prospectively informed and warned about the deception involved in the study, the subject still may not know sufficient details about the deception. This kind of “global” consent to the deception in the abstract is not sufficient to avoid dishonesty. Of course, Wendler and Miller acknowledge this limit by saying, “participants never know everything there is to know about any study” (Wendler and Miller 2008, 321). But that doesn’t mean that there is no way to enhance the validity of informed consent by adding more specificity without undermining the scientific validity of the study.

Let me suggest some possible strategies that can address this issue. One is *disjunctive informing*, which is to ask for consent based on disjunctive information. In deceptive placebo studies, to ensure unbiased reporting of the medication’s effects, the researchers may tell all the subjects that they will receive active medication when half of them would receive a placebo (e.g., a sugar pill) instead. This is a clear case of deception. We may make it more honest by giving disjunctive information to the participants rather than deceiving them. For example, a researcher may say to its subject, “This pill may be *either* active medication *or* a sugar pill. Would you take it for the research?” If the subject says yes, then it can be a way to get sufficiently informed consent without informing the subject about the details that may undermine the scientific validity of the study results. For the subject is informed about the possible disjuncts she may be choosing, although she is not informed about which one it will be. In this way, the researcher can study the effectiveness of the potential medicine without worrying about biased reporting, since the disjunctive information given here would not make a difference in the subjects’ belief about the effectiveness of what they take a dose of.

The strategy of disjunctive informing has the following merits. First, offering disjunctive information can give the subject a chance to examine if she is fine with each disjunct without revealing which one will actually be applied to her. In our example, the subject would give consent only if she is okay with either the potentially effective medicine or a sugar pill. This is an advantage compared to the case in which the subject is not informed about the list of substances that can possibly be applied to her in the research. Second, disjunctive informing can ensure unbiased reporting of effects without involving any deception. For it does not involve intentionally causing the subjects to believe what the researcher does not believe to be true. While some subjects may form false beliefs about what they are taking, that is unlikely to be caused by the disjunctive information offered by the researcher.

Another strategy is *domain specification*, which is to specify the domain of deception, rather than its contents. That is, we can respect the subject RND by specifying the domain of the matter about which the deception in question may occur, to the extent that it leaves the possibility of effective deception. If the subject *waives* her RND in the specific domain in question by giving consent, deceiving her in that domain does not necessarily manifest dishonesty, since it would no longer be a way of disrespecting her RND. It is possible to consent to waive one’s RND in a specific domain without waiving it in other domains. Consider a case of a poker game. When we play a poker game, we (at least implicitly) consent to waive our RND in a specific domain, namely, about what cards one is holding. This is why “bluffing” about one’s cards does not count as dishonest in such a context. However, if a player deceives another in some other unconsented domain, then it does manifest dishonesty. For example, sneaking an extra card in one’s sleeve is a dishonest kind of deception since no players have waived their RND in that domain.

Similarly, the researcher can specify the domain in which deception may take place in the study and ask for consent to participate. To apply this idea to the placebo-controlled research, the researcher may ask: “You may be deceived *concerning the degree of effectiveness* of the pills provided, but there will be no deception involved in other aspects of the study. Would you still participate?” Another example is empirical research investigating the gap between perception and reality, where deceptive visual or auditory stimuli are used to study how the brain processes conflicting information and constructs perceptions. In such a study, the researcher may inform that there might be deceptive elements among the perceptual stimuli provided in the study and then ask for consent (see, e.g., Mouatt et al. 2023).

Of course, it would require wisdom to strike the right balance between abstractness and specificity so that the subject is informed enough to give valid consent and the study results remain reliable. Still, if the researcher strives to find the right spot, she would be able to conduct more honest research without losing scientific validity. The strategies suggested above has focused on the cases of deceptive research in which the researcher tries to acquire at least some form of informed consent from the participant. Of course, there still are many cases of deceptive research that cannot be covered by the methods suggested above. Even the strategies of disjunctive informing or domain specification may undermine the scientific validity of deceptive studies such as mystery shopping experiments, a study in which a researcher pretends to be another participant, or covert research where the participants do not even know they are part of the research.

The problems of dishonesty involved in such studies may need separate discussions. Also, some cases of human subject research that involve dishonesty may still be morally justified by other ethical considerations such as the urgency of new drug development. However, the main aim of this paper has been to identify the potential elements of dishonesty that may be involved in human subjective research and find possible ways to minimize such elements without undermining the scientific validity of the research.

## Conclusion

In this paper, I have raised some ethical concerns that arise from the deception involved in human subject research and examined possible solutions. An ethical researcher would disclose all relevant information to the participants and ensure that the study is conducted in a transparent manner. But the practice of respecting the participants by asking for adequately informed consent may be undermined if the research requires deception for its scientific validity. I have examined the possible ways to make an honest research plan and suggested several approaches to render the research more honest.

My aim has been to analyse the ethical issues related to deceptive human subject research in terms of honesty, which I understand as respect for the right not to be deceived. There are different ways to avoid dishonesty in human subject research. First, we can try to minimize the element of deception if possible, since it is likely that deception involves dishonesty or disrespect for RND. Second, if it is hard to avoid deception (e.g., for scientific validity), we should make sure that deception involved in research does not manifest the vice of dishonesty. For example, if one consents to waive one’s RND in a specific domain, as in the case of poker players, then deception in that domain would not manifest dishonesty. Finally, given that honesty is not the only virtue that makes demands, some other virtues may require us to do something that would manifest dishonesty in a normal situation. In such a case, the researchers should make sure that the deception involved in the research (plan) is justifiably overridden by the demands of other virtues such as benevolence. In such a case, it would be hard to say that the research is particularly honest. But given that it is justified by other moral demands, it can at least be morally permissible, all things considered. Thus, we can say that this kind of research is neither honest nor dishonest.

Human subject research will remain an essential component of scientific advancement and provides valuable insights. Some cases of such research may require deceptive elements to obtain correct and objective data about how human participants react. There might be some cases where deception is justified by the significance of the expected scientific discovery. However, it would still be ethically important to make our research plan as honest as possible. I have tried to show what makes a research plan dishonest, why it is important for research to be honest, and how to improve a given study’s degree of honesty. The implementation of deception in human subject research should be approached carefully, and researchers (as well as IRB members) should be mindful of the importance of honesty or respecting the participants’ RND.

## Data Availability

Not applicable.

## Footnotes

1 Let me add a note on the notion of “informed consent.” I believe “informed consent” is a matter of degree. Some cases of consent may be sufficiently informed while others are not. It is not an all-or-nothing matter. There can be informed consent even in a deceptive study. The question is whether the consent is made based on sufficient relevant information. Consider the following types of cases. First, what would be taken as insufficient information in an ordinary situation can be “sufficient” in the sense that it is sufficiently justified by some other values of the research as a whole. Second, some studies may give false or misleading pieces of information rather than omitting some relevant information. In such case, we can say that *informed* consent was given in some broad sense, and then we should ask if the false or misleading information can be justified. The whole point of this paper is to show how we can make research as honest as possible, rather than to narrowly focus on whether “informed consent” is given or not.

2 In Um (2024), I explain what RND means and why respect for RND is at the core of the virtue of honesty in more detail, but here it would suffice to briefly sketch it to show how it can be applied to the case of human subject research.

3 Of course, this does not exhaust considerations of autonomy. The point here is that the consideration of subject’s willingness here may disrespect her RND without necessarily disrespecting her right to autonomy. I thank the anonymous referee for making this point.

4 For more on the difference between RND and the right to autonomy, see Um (2024).
